# Perspectives on integrating family planning and nutrition: a qualitative study of stakeholders

**DOI:** 10.1136/bmjgh-2024-015932

**Published:** 2025-01-06

**Authors:** Sachin Shinde, Uttara Partap, Nazia Binte Ali, Moussa Ouédraogo, Yohana Laiser, Iqbal Shah, Wafaie Fawzi

**Affiliations:** 1 Department of Global Health and Population, Harvard School of Public Health, Boston, Massachusetts, USA; 2 Nouna Health Research Center, Nouna, Burkina Faso; 3 Africa Academy for Public Health, Dar es Salaam, Tanzania, United Republic of

**Keywords:** Qualitative study, Nutrition, Global Health, Health services research

## Abstract

**Background:**

Limited information is available on the value of integrating family planning and nutrition services to improve related outcomes among women of reproductive age and effective approaches to achieve this. This study aimed to ascertain the perspectives and experiences of global and regional stakeholders about integrating family planning and nutrition services, examine facilitators and barriers and identify opportunities and considerations for integration.

**Methods:**

We conducted semistructured interviews with 34 global and regional stakeholders in family planning, nutrition and related domains. Participants were identified through purposive sampling. Interviews were conducted virtually, recorded and transcribed. Data were analysed using thematic analysis.

**Results:**

Stakeholders considered the integration of family planning and nutrition services potentially valuable given the biological links between family planning and nutritional status, and potential practical benefits including increased service coverage, reduced burden on beneficiaries to access services and increased cost-effectiveness of service delivery. Integration was commonly described within the context of comprehensive health service packages, with integration models encompassing health systems strengthening, life course and multisectoral approaches. Facilitators and barriers included systemic and structural, resource-related and contextual factors. The need for more robust evidence to support integration and identify effective and cost-effective integration models was emphasised.

**Conclusions:**

Integrating family planning with nutrition services and both with other health services directed towards women of reproductive age and their children may offer greater value in improving health and related outcomes, as opposed to siloed approaches. Further evidence quantifying benefits and highlighting the effectiveness of such integration strategies is key to informing future programmatic efforts.

**WHAT IS ALREADY KNOWN ON THIS TOPIC**

- Generally, family planning, nutrition and related health and non-health services have been addressed separately through vertical programmes, lacking comprehensive cross-sectoral dialogue at the levels of policy, strategy, funding or programme implementation. Limited evidence regarding successful strategies for achieving this integration hampers future initiatives.

**WHAT THIS STUDY ADDS**

- Through qualitative interviews with global and regional family planning and nutrition stakeholders, our study highlighted the potential value of and different ways of integrating family planning and nutrition services. Various enablers and barriers, including systemic, resource-related and contextual factors, were identified. Our study also emphasised the necessity for more robust evidence on integration and identifying effective and cost-effective models.

**HOW THIS STUDY MIGHT AFFECT RESEARCH, PRACTICE OR POLICY**

- This study emphasises the increasing recognition of the importance of establishing connections between nutrition and family planning programming for women of reproductive age. It underscores the necessity to account for contextual factors and employ innovative approaches such as life-stage interventions, leveraging existing platforms and service providers, and mHealth techniques in designing and delivering integrated family planning and nutrition services to improve both individual and community health and well-being.

## Introduction

 In women of reproductive age (15–49 years), substantial challenges with undernutrition persist, affecting around 10% of women and 25% of adolescent girls (10–19 years), with anaemia affecting millions globally, compounded by prevalent deficiencies in iron, zinc or folate, and rising overweight and obesity in low-income and middle-income countries (LMICs).^1^,^4^ Similarly, nearly half of all pregnancies (or 21 million each year) are unintended because of not using family planning.^5^ Optimal nutrition and family planning are essential to the health and well-being of women of reproductive age (hereafter referred to as women), with benefits extending to their children. Malnutrition in women, including both undernutrition and overweight or obesity, is associated with multiple adverse consequences including poor quality of life and increased morbidity, poor labour force participation, productivity and economic potential, and adverse pregnancy outcomes and intergenerational health outcomes.^46^,^8^ Having early, short-spaced and numerous pregnancies has similar consequences and slows the healthy growth of adolescent mothers.^9 10^

In addition, family planning is understood to positively impact the nutritional status of women and their children through multiple pathways. This includes delaying age at first pregnancy and thereby ensuring that available nutritional resources are directed towards optimal growth and development during adolescence^11^; enabling girls and women to attain a higher level of education and socioeconomic status, thereby increasing their potential to access a healthy diet; increasing interpregnancy interval and preventing maternal depletion^6 7 12^; decreasing family size resulting in increased allocation of nutritional resources to mothers and children^13^; and, reducing menstrual blood loss through direct hormonal effects.^14^ Additionally, by enabling optimal breastfeeding for each child, family planning contributes to improved child nutrition.^15^ However, a recent systematic review and meta-analysis examining the relationship between family planning methods and nutritional outcomes among adolescents and women of reproductive age in LMICs revealed limited robust evidence linking family planning use to various maternal nutrition outcomes. The findings suggest weak support for certain family planning methods, particularly hormonal intrauterine devices (IUDs), in improving haemoglobin levels and potentially reducing body mass index (BMI).^16^ This highlights the need for further research to critically evaluate the potential impact of modern contraceptive methods on the nutritional outcomes of women in LMICs.

Strategies to improve nutrition and achieve optimal number and timing of pregnancies are therefore key to achieving the best outcomes for women and their children. Despite this, multiple challenges remain in ensuring access to adequate family planning and nutrition, particularly in LMICs. This can be due to various factors, including geographical remoteness, poverty, cultural barriers and limited empowerment of women, and weak health systems.^17 18^

Integration of health and related services across domains is recognised as a potentially important strategy to improve the reach and impact of these services for women. For example, nutritional counselling and supplementation as part of antenatal and postnatal care to improve maternal and infant health is encompassed in global recommendations and country policies and programmes.^19 20^ Furthermore, some studies have examined the integration of family planning services with maternal and child health, immunisation, HIV/AIDS and other STIs, and intimate partner violence prevention programmes.^14^ There remains, however, limited knowledge about how to integrate family planning and nutrition services effectively and sustainably, including as part of such programmes.^19^,^21^ To inform further research and action, exploring perspectives and experiences of global and regional stakeholders in family planning and nutrition about the feasibility and benefits of such integration is necessary. This study aimed to understand the views of global and regional nutrition, family planning and related domain stakeholders about the potential value of and ways to integrate family planning and nutrition, examine key facilitators of and barriers to such integration efforts and identify future opportunities and relevant considerations for designing and implementing such efforts.

## Methods

### Study design and participants

We conducted a cross-sectional qualitative study using semistructured in-depth interviews with global and regional stakeholders in family planning and nutrition. This investigation enabled us to explore the experiences and perspectives of global and regional stakeholders engaged in integrating family planning with nutrition, or with other health and health-related domains such as maternal, neonatal, and child health, postabortion care, sexual and reproductive health, adolescent health, food insecurity, social protection, and humanitarian aid.

Study participants were identified through multiple channels. First, we leveraged our existing network—The Africa Research, Implementation Science, and Education Network (https://www.aaph.or.tz/activities/arise-network)—which includes global and regional experts engaged in global health and nutrition research. This network consists of professionals with expertise in family planning, nutrition, sexual and reproductive health, HIV/AIDS, health systems strengthening, and service integration, enabling us to efficiently identify key stakeholders.

Second, we also reviewed websites of organisations involved broadly in family planning, nutrition, reproductive health, and maternal and child health programmes. Organisations were identified through a combination of our scoping review, which sought evidence on prior programmes that integrated family planning with relevant services, as well as recommendations from interview participants. These organisations were chosen based on their prominent roles in shaping policies and implementing family planning and nutrition initiatives and spanned a wide range of sectors, including government agencies, academic institutions and leading international health organisations such as WHO, UNFPA, the Food and Agriculture Organization, World Bank, Population Council, Marie Stopes International and Population Services International, as well as regionally focused non-governmental organisations (NGOs) in sub-Saharan Africa, South Asia and South America—regions that are heavily involved in global efforts to integrate family planning with nutrition and broader health programmes.

We contacted 64 stakeholders from global and regional organisations via email and successfully conducted interviews with 34 of them. Although three additional stakeholders agreed to participate, they were unavailable during the data collection period. As per our protocol, we sent two reminder emails to the 27 non-respondents but did not receive any further replies.

### Participant recruitment and data collection

Data were collected between April and August 2023. One-to-one semistructured interviews were conducted using the Zoom platform. All interviews were conducted in English by two authors (SS and IS) trained in qualitative research. Interviews lasted between 45 and 60 min. Each interview was recorded and transcribed using Zoom transcription function. Zoom transcripts were reviewed against audio files, with necessary modifications annotated.

The interview guide covered (1) the participants’ organisational and individual programmatic expertise and experiences in the fields of nutrition, family planning, sexual and reproductive health and related health and non-health domains; (2) their experiences pertaining to the integration of family planning with nutrition and related services, including motivations, lessons learnt, barriers and challenges and (3) their insights into the current global discourse, promising practices, opportunities, and considerations and recommendation for future research and practice (online supplemental file 1). The interview guide was pilot tested with the first two participants and necessary adjustments were made; the final analysis includes the two pilot interviews.

### Analyses

We used a grounded theory approach to thematic analyses, encompassing deductive and inductive coding.^22^ With this approach, both inductive and deductive qualitative data analysis were combined. The themes covered in the interview guide provided an a priori framework (deductive) for analysis, whereas perceptions and views expressed by participants allowed the identification and refinement of critical themes grounded within the data (ie, inductive).

The following steps were employed for data coding, analysis and interpretation. First, two authors (SS and UP) read and familiarised themselves with the data. In the second step, these two authors selected a subset of three interviews and categorised parcels of data using open codes and annotated notes. Subsequently, they identified and compiled codes into potential themes to create a codebook guided by the codes and original research questions. In the fourth step, the codebook was independently applied to three randomly chosen interviews.^23^ After coding, any instances where consensus was not reached were addressed through discussion and resolution. This stage involved cycles of inductively elaborating themes from the data and deductively applying them to the data. This process helped ensure the coherence of data within each code and maintain clear distinctions between codes. This framework was refined iteratively through discussions until all authors were satisfied that it accurately reflected the data. In the fifth step, three authors (SS, UP and NBA) applied the codebook to all interviews, creating a matrix framework containing verbatim and summarised data from the transcripts in Microsoft Excel.

To ensure the reliability and consistency of our coding process, a mixed coding approach was employed. 25 of the 34 total interviews were independently coded by at least two authors to establish inter-rater reliability (90% agreement calculated as the total number of agreements divided by the total number of comparisons). Due to time constraints, the remaining nine interviews were coded by a single author and subsequently reviewed by another coder to maintain data quality. During the concurrent process of interviewing and coding, the authors met weekly to compare new data with earlier transcripts to ensure the resulting matrix provided a detailed and accessible overview of data within each theme and subtheme, as reported by the study participants. It also facilitated the grouping and comparison of existing constructs related to the integration of services, especially focusing on family planning with nutrition and other relevant services. Finally, the authors used the completed matrix framework for data exploration by both theme and respondent type. This approach facilitated the development of a comprehensive description of each theme and subtheme, enabling the identification of patterns, positive and negative deviants, and excerpts to support themes and subthemes in the data.^22 23^

### Patient and public involvement

No members of the public were involved in this research.

## Results

### Participant characteristics

We interviewed 34 stakeholders, including 11 representatives of international NGOs, 8 from academia, 7 from United Nations agencies, 5 from donor agencies and 3 from civil service organisations or federations (table 1). 23 participants were female.

**Table 1:** Key characteristics of study participants, n (%)

Participants	n	%
Sex		
Male	11	32.4
Female	23	67.6
Organisation type		
UN agency	7	20.6
Civil service organisation or federation	3	8.8
International non-governmental organisation	11	32.4
Donor or funding agency	5	14.7
Academia, including independent researchers	8	23.5
Regions		
Africa	9	26.5
Asia and Southeast Asia	7	20.5
Europe	6	17.6
North America	8	23.6
South America	4	11.8
Total	34	

The regional representation of the participants included nine from institutions based in Africa, eight from North America, seven from Asia and Southeast Asia, six from Europe and the remaining participants from South America. We found that the characteristics of the 30 participants who were approached but did not participate were broadly similar to those who did.

While the study focused primarily on family planning and nutrition, most participants described these services as being an integral part of a broader approach that integrated multiple services for women, children and adolescents. From the interviews, multiple themes and subthemes emerged, which are summarised in table 2, and presented below.

**Table 2:** Summary of the results by theme

Theme	Subtheme	Key results
Philosophy of and conceptual frameworks for integration	Perspectives on rationale for integration of family planning with nutrition and other health services	Described as logical, because of the interconnection between family planning and nutrition services and the health implications they have for women and their children: Short interpregnancy intervals and early pregnancy can increase the risk of undernutrition in mother and consequently have adverse health outcomes for both the mother and the infant. Short intervals between pregnancies and large family sizes can lead to insufficient resources for maternal and child nutrition and care. Early marriage and adolescent pregnancy can hinder education, employment opportunities and social mobility, negatively affecting nutrition. Contraceptive use can affect menstrual blood loss and the risk of anaemia. Human rights and empowerment principles were mentioned as part of the rationale to integrate family planning and nutrition within comprehensive healthcare to ensure quality care. Reduced access to services linked to gender inequality, women’s lack of agency and broader influences including environmental factors, requiring an integrated approach. Integration approach enhances coverage of services, reduces unmet needs and is seen as cost-effective and efficient; need for further evidence underlined.
	Models and approaches for integration of family planning, nutrition and other services	Health systems strengthening Efforts aimed at strengthening health systems through sustainable infrastructure and capacity building. Examples included multiple services provided at one facility or community visit by one or more providers, coordinated services under one programme and referrals within and between facilities. Life course approaches Focused on life cycle stages, spanning prepregnancy, pregnancy, adolescence, reproductive years and postreproductive years. Several community platforms and non-health professionals recognised for delivery of integrated services. Multisectoral approaches Highlighted programme integration encompassing multiple sectors (eg, health, education, social protection, climate crisis) acknowledging their interconnectedness. Stressed the significance of involving individuals, groups and institutions in the entire process of integration, with government-led action plans. Approaches to engagement included provision of resources, information, capacity building and leadership training, and financial and technical assistance.
Facilitators of and barriers to integration	Systemic, organisational and infrastructural facilitators and barriers	Strengthening primary healthcare provision and health systems seen as crucial for enhancing success of integrated approaches. Vertical and siloed programming noted to hinder integration whereas integrated approaches facilitate execution of successful efforts. Government willingness and leadership identified as a key to endorse, fund and manage integration efforts while the absence of coordinated leadership seen as a barrier. Clear budgets, reporting systems and accountability mechanisms identified as facilitators of integration.
	Resource-related facilitators and barriers	Although seen as cost-effective, integration efforts require significant infrastructure changes, which presented challenges in resource-constrained settings. Shortage of supplies and logistical challenges believed to erode trust and lower healthcare facility performance. Siloed funding and donor reluctance to long-term investments hindered integration efforts. Investment in human resources through capacity building, mentoring, and incentivisation promoted integration, while shortage of trained staff and overburdening of existing staff posed barriers to integration.
	Contextual facilitators and barriers	An enabling policy environment and clear population-focused mandate facilitated integration. Conservative community perceptions and regulations related to family planning hindered acceptance of integrated services. Engagement with religious and community leaders supported integration. Global challenges like pandemics and economic and political instability disrupted integration efforts.
Gaps and opportunities for integration of family planning and nutrition services		Need for greater evidence for integrating family planning and nutrition services, with specific need for higher-quality data supporting integration rationale, design and programme success. Generating evidence on macrolevel issues like poverty, gender equality, empowerment, and climate change with family planning and nutrition would build the case of investment in upstream drivers. Data required on benefits to governments, service providers and clients to justify investment in integration—including demonstrated reduction in costs, improved client uptake and favourable cost–benefit ratios. Need for the implementation science research documenting the added value of integrated approaches to advance the integration agenda.

### Philosophy of and conceptual frameworks for integration

Two subthemes, encompassing perspectives on the justification for integration and various models and approaches to integration, surfaced within the overarching theme of the philosophy of and conceptual frameworks for integration.

Integration of family planning and nutrition services was described as ‘logical’ and a ‘no brainer’, given their interconnected nature and impacts on women and their children’s health. Among the reasons given were (1) early pregnancy and short interpregnancy intervals can put mothers at risk of undernutrition, resulting in adverse health outcomes for the mother and also leading to poor neonatal outcomes (such as low birth weight) which are associated with persisting undernutrition in childhood; (2) short interpregnancy intervals and larger family size may result in inadequate resources for nutrition and care of children; (3) early marriage and pregnancy among adolescents can adversely impact their completion of education and participation in the labour force, as well as their social mobility—all of which may have adverse impacts on nutritional status and (4) contraceptive use such as oral contraceptive pills, hormonal IUD, implants and injectables may significantly reduce blood loss through menstruation and therefore the risk of anaemia.

From a human rights-based and empowerment-based perspective, the integration of family planning and nutrition services was also deemed essential. Participants noted the right to quality healthcare and highlighted the importance of comprehensive care that encompasses various health-related needs, including family planning and nutrition services. Providing multiple services during a single visit or point of contact was recognised as an effective means of reducing the burden on beneficiaries who may face travel and distance barriers. Furthermore, the participants linked reduced access to family planning, nutrition and other health services to several factors, including gender inequality and women’s lack of agency and empowerment, in turn, influenced by social, economic and environmental factors such as conflict, climate change and food insecurity. Integrating approaches including targeting these upstream drivers was recognised as essential for achieving the best outcomes for women, children and their families. One participant mentioned,

…researchers and policymakers do not talk about macro trends that are driving the relationship between family planning needs and nutrition a little bit more [severely] in the future…we need to look at how the climate crisis is driving food insecurity and therefore influencing pregnancy and fertility outcomes, which dictates nutrition…in our work, we approach it through climate, livelihood and empowerment.

Integration and provision of multiple services including family planning and nutrition, particularly at the community level, was recognised to increase coverage by reducing missed opportunities, thereby reducing unmet needs. Integrating approaches across health and non-health domains was also mentioned as more cost-effective and efficient, particularly in the long term. Some participants, however, emphasised the need for more evidence about factors such as effectiveness, efficiency and cost-effectiveness when integrating services within the context of existing systems and infrastructure. Given limited human and financial resources in LMICs, this was noted as a critical factor.

### Models and approaches for integration of family planning, nutrition and other services

From a functional ‘one-stop shop’ to broader integration of health services, several examples were shared of how family planning was integrated with nutrition, maternal, neonatal, child and adolescent health, and other health and non-health services. Three broad and overlapping categories of integration approaches emerged: (1) health systems strengthening, (2) life course approaches and (3) multisectoral approaches.

#### Strengthening the health system approach

Often described as reaching the last mile, participants mentioned efforts to strengthen equitable and comprehensive health systems overall and in remote and marginalised communities. This entailed developing sustainable infrastructure and building capacities of health facilities to plan, deliver, monitor and evaluate a comprehensive package of services, including family planning and nutrition. Integration models described were often opportunistic, aiming to leverage all contact points to identify and address health, nutrition and family planning needs, to ensure ‘zero missed opportunities’. Different methods of service integration were shared. These included (1) multiple providers providing services within a single setting during the same visit; (2) service provision at a single contact point by a single provider such as a nurse or a community health worker; (3) coordination of services under one programme by different providers at different time points and/or locations and (4) referrals between facilities or between departments within the same facility during the same visit or across multiple visits. Top-down approaches involved capacity building through cascade training for service providers on integrating services. For bottom-up approaches, participants described mixed-methods formative research to design and implement context-specific and service-specific programmes before implementing an effort at scale. A significant part of both approaches involved building connections between government ministries and departments and stakeholders who could support policy development, programme implementation, financing, resource allocation, and monitoring and evaluation. Public–private partnerships were also recognised as potentially key to promoting the expansion of services. One participant shared,

We developed the model called ‘zero or no missed opportunities’, using every facility contact opportunity, to cover women’s and their babies’ health needs in the critical antenatal and postnatal periods. This model is about delivering a comprehensive package of essential and lifesaving services across four main entry points … at facilities during antenatal to postnatal periods. So we have a package of essential services at antenatal care, at delivery point, at postnatal care, and at infant care, including family planning, nutrition, and cervical and breast cancer screening—so organizing services in one stop shop with a multi skilled provider, or co-location with several complementary providers, referring women from one point of care to another in the same facility during the same visit, and at the same cost.

Family planning and nutrition services delivered as part of integrated programmes included behaviour change communication for family planning demand creation, family planning counselling and commodities, screening for nutrition and sexual and reproductive health issues, nutritional supplements, and nutrition education and counselling. Integrated services were provided by front-line workers (community health workers, female health workers, health extension workers and health volunteers), doctors, nurses, midwives, pharmacies and other private providers. In addition, elected local and community leaders were recognised as key facilitators of integration efforts. The key platforms used for integration were health facilities (including subcentres, clinics and health extension centres), mobile clinics, workplaces, digital media and homes.

#### Life course approach to integration

Many participants emphasised the importance of life course approaches for family planning, nutrition and reproductive health, during critical developmental phases, such as prepregnancy, pregnancy, adolescence, reproductive years and postreproductive years. One participant shared,

We intervene when girls are 14 to16 years old because that is when most of the girls drop out of school, especially from poorer communities. Another set of intervention is for girls when they are 16 to19 years old because that is when early marriages take place. We provide information on contraception, ensuring family planning choices along with maternal healthcare…and then across these life stages we intervene to change social norms specifically around prevention of domestic violence.

Participants strongly recommended investing in adolescents since health status and skills acquired during adolescence have a profound impact on life course trajectories, including for nutritional and other outcomes. Several evidence-based approaches were shared to improve multiple aspects of adolescent health. Nutrition-specific and nutrition-sensitive interventions including school feeding, as well as comprehensive sexuality education, were among them, as were non-health interventions, such as life skills education, gender equality and empowerment approaches, and climate change adaptation. Schools were unanimously identified as important platforms to integrate services. Participants mentioned that keeping adolescents in school longer has a positive impact on their health and nutrition, educational status, workforce participation and economic productivity. Some also indicated the importance of continuing to focus on community-based platforms while others reported the use of digital media platforms to reach adolescent populations with messages regarding health and well-being. Furthermore, participants stressed the importance of developing evidence-based adolescent-friendly health services. In addition to health providers, teachers, peers, community leaders and front-line health workers were identified as providers for promoting health and well-being. Participants also discussed the importance of and shared examples of incorporating life course approach education into nurse, doctor and teacher training.

#### Multisectoral approach to integration

Several participants supported the idea and offered examples of how to incorporate family planning, reproductive health, nutrition, food insecurity, social welfare and protection, and the global climate crisis into their programmes, emphasising the connection between these sectors. The most cited examples of integration of family planning, nutrition and other domains in this context were welfare or social protection programmes targeting adolescents and women. There was recognition that women and children’s health, nutrition and family planning interventions could be included in such programmes to maximise their impacts on beneficiaries. Multiple levels of action were typically involved in these programmes, including building skills, cash transfers, provision of services and commodities, and opportunities at the individual, household, and community levels. One participant shared,

…the communities are in extreme poverty with high security risks and climate vulnerabilities such as frequent droughts. So, we target the adolescent girls with a mentoring programme with nutrition [education] component and a cash transfer programme…this combination has a major impact on pregnancy outcome six years later.

Overall, participants stressed the significance of involving individuals, groups and institutions in planning, developing, implementing and evaluating integrated family planning services with nutrition, health and non-health programmes. Integration efforts were typically government led and regionally owned, involving diverse approaches such as providing information, tools, leadership capacity, and financial and technical assistance. Some highlighted the importance of open communication and collaborative problem-solving. One participant mentioned,

We organize a separate workshop for the development partners to present their respective workplans and they are later reflected in the government registers…this helps [in improving] accountability.

### Facilitators and barriers to integration from programmatic experience

Participants shared several facilitators of and barriers to integrating family planning and nutrition services. These are broadly presented in three categories: systemic, organisational and infrastructural; financial and human resources and contextual facilitators and barriers.

#### Systemic, organisational, and infrastructural facilitators and barriers

Progress towards vital primary healthcare provision and strengthening health systems was noted as a critical facilitator for enabling integration of family planning, nutrition and other programmes. Most participants believed that strengthening lower-level service delivery points such as community clinics, health posts and outreach extensions than tertiary level points would facilitate integrated service delivery since more women would be able to reach them more easily. In contrast, vertical funding streams for sexual and reproductive health, maternal and child health, and siloed technical programming at the Ministry of Health were thought to undermine service integration.

The critical leadership role of the representatives in the government and officials in various ministries was believed to be an important facilitator in integration of family planning with other services. Their involvement was acknowledged in providing endorsements, allocating funding, communicating with funding agencies, and planning and managing implementation. On the other hand, lack of coordinated leadership was considered an impediment to family planning integration with other services. One participant shared,

A few challenges that most countries face are weak leadership, limited ownership of integrated programmes and over reliance on the donors. I have seen that countries that have strong leadership and strong sense of ownership over the programmes, their donors try as much as possible to support the government.

It was also noted that clear lines of budgets, reporting systems and creating accountability mechanisms to address problems were critical facilitators to integrate services. Participants also expressed that funding multiple partners rather than one agency or ministry, investing in the development of local leadership capabilities, strengthening financial management and good practices in transparency, reporting and auditing to ensure accountability to the public helps combat corruption and facilitate integration.

A lack of appropriate infrastructure was also identified as a barrier to the integration of family planning and nutrition services in LMICs. For example, the lack of space in lower health units and outreach services poses a severe challenge to discussing family planning needs confidentially.

#### Financial and human resource-related facilitators and barriers

Participants indicated that integration of family planning, nutrition and other services could eventually be less costly and more effective, but it may require major and expensive changes to current infrastructures. Resource and logistical challenges leading to stock-outs of essential commodities and related equipment particularly for family planning services were also perceived to be obstacles to integrated services, leaving clients uncertain and mistrustful, and healthcare facilities unable to reach their targets. The COVID-19 pandemic was believed to have at least temporarily increased supply chain barriers in certain cases. Despite recognition of cost efficiency in the longer term, a few participants also raised the issues of lack of initial financial and other resources to achieving an integrated health system. It was noted that donors were less inclined to fund activities showing less immediate impact due to the long-term nature of health systems strengthening. A participant said,

Building the capacity of systems is a long-term agenda, and you cannot show results instantly… that’s a big problem… the donors have accountability for their taxpayers, and they want instant results with demonstrable impact. So, the process indicators do not do the trick anymore. This is a contradiction because integrated programmes require time to show impact.

Funding was identified as a key factor influencing integration efforts. Integrated programmes were recognised as being hindered by low domestic financing. Separate funding arrangements were also said to prevent synergy, reduce the efficiency of budget allocations and planning of services, and increase administration and implementation costs. Some participants noted that external funding was siloed and focused on vertical programmes or singular domains, providing little possibility for integration. Others indicated that the funding landscape was changing, with donors beginning to fund integrated programmes or mandating that agencies collaborate across domains. Some funding agency representatives said that multiyear funding cycles facilitated integration efforts to progress.

Adequate investment in human resources was identified as another important factor. To provide a quality integrated family planning and nutrition service, it was deemed critical to strengthen capacities and build competencies among all cadres of service providers. Providing formalised in-classroom and on-the-job training, and mentorship and supervision to service providers, managers, and officials was identified as a crucial factor in facilitating integration at all levels. Promoting a positive attitude towards integration efforts among healthcare providers and government officials was identified as another key enabler, driving ownership of the integrated processes and aiding service providers in understanding what and how to integrate without bias. Many commented on the importance of appropriate remuneration and sufficient incentivisation as an important factor in motivating healthcare providers to maintain enthusiasm for integration and quality of services. A participant explained,

…investing in the staff to have the culture of critical thinking is crucial, because ultimately, it is the staff, the health workers, the people who implement such integrated programmes on the ground.

Inadequate training of human resources to deliver health services and insufficient coordination among different service providers were identified as barriers to integrating family planning and nutrition services. In lower-level health units, this barrier was perceived to cause fatigue and burn-out in a few staff members. Long wait times and substandard care were also attributed to a shortage of service providers. It was noted that increasing the number of tasks or responsibilities of a single health worker as part of integration, especially by adding more time-consuming services such as nutritional counselling, was not sustainable and would affect the quality of service. One participant mentioned,

The health workers have limited time to spend with each woman … it is difficult to provide proper nutrition education and counseling with the limited time they have.

#### Contextual facilitators and barriers

An enabling policy and programme environment with a clear mandate for addressing the unmet needs of the population was identified as a facilitator in the integration of family planning and nutrition services. On the contrary, the perceptions and regulations surrounding family planning and fertility, particularly in conservative communities, were noted as an important barrier to integration, with issues becoming even more sensitive in the context of targeting adolescents or unmarried youth. For example, participants noted the existence of regulations surrounding whether and what contraceptives could be provided in schools, or what specific contraceptive methods nurses could provide.

The history of family planning as a form of population control and cultural values around women’s fertility was perceived to decrease the receptivity to integrated interventions that included components of family planning. Some participants indicated that such perceptions hindered or could hinder the ability to integrate family planning services with more widely accepted services including nutrition counselling and supplementation or immunisation programmes, as programme leaders were concerned that integration would lower uptake of the base services. On the other hand, a few participants argued that adding family planning services to other interventions helped to improve coverage of family planning services by making it a less visible or obvious component. Engaging with community and religious leaders in conservative societies was identified as a facilitator of integrated programmes.

Aspects of the wider global and international context were also identified as important enablers or barriers. Being in alignment with national and international movements in family planning and nutrition helped local integration efforts, particularly in terms of setting national agendas, targets and action plans. On the contrary, developments such as the COVID-19 pandemic, economic instability and political strife were recognised as key challenges that disrupted integration efforts and affected population needs.

### Gaps and opportunities for integration of family planning and nutrition services

Participants identified several gaps in the available evidence and described multiple opportunities to advance the integration of family planning and nutrition services. Future opportunities for integration identified by participants broadly mirrored recent or ongoing efforts described by others in terms of integration models, target populations, providers, platforms and services, with many continuing to emphasise multisectoral efforts targeting key points in the life course and involving health systems strengthening.

Central to responses was the need for higher-quality evidence regarding the integration of family planning and nutrition. This included evidence to support a rationale for integration, to inform the design of integrated strategies, and to demonstrate the success and feasibility of integrated programmes. Although there was acknowledgement in principle of the epidemiologic pathways through which family planning affected nutritional outcomes, participants noted that actual evidence documenting these pathways remained lacking. Such evidence was noted as key to convincing donors and governments to invest in a comprehensive approach integrating the two domains and guiding them on how to allocate resources. Specifically, the need was identified for stronger data quantifying the effect of the use of family planning on nutritional status among women. In addition, participants suggested the need for evidence regarding intergenerational effects, including the impact of family planning on maternal and child nutrition, morbidity and mortality, with a view to understanding effects on human capital. Such data were recognised as valuable to inform models and tools to guide governments on resource allocation to improve population health and productivity. One participant expressed,

We still don't see the evidence in terms of impact of family planning on child stunting, wasting or any of the other indicators…we can't include family planning [into nutrition programing] even if we want to, because that evidence is not there.

Participants also noted the need to connect this evidence with wider, macrolevel issues including poverty and livelihoods, gender equality and empowerment, and climate change. Evidence outlining the specific impacts of such issues on family planning and nutrition would help to build the case for further investment in interventions mitigating these upstream drivers, with benefits from such interventions expected to cascade to family planning access and use and nutritional status among women and adolescents.

Evidence regarding benefits to governments, service providers and clients was also identified as important to justify investments in integration. Specifically, it was deemed critical to demonstrate that for integrated versus separate or vertical programmes: (1) human and financial resources required to deliver services would be reduced and/or (2) there would be a net increase in benefits to clients, such as increased uptake of all services and lowered costs to access services. Others extended this idea, pointing towards the need for conducting comprehensive cost–benefit assessments. Overall, it was stressed that such evidence regarding the success of tested models was essential to drive the funding and adoption of integrated programmes. One participant outlined,

We have to show that there are benefits in integration but there are very few studies that have shown that this works.… it’s incredibly rare that people bother to do that kind of study.

While no single model for integrating family planning with nutrition and other services was identified, participants highlighted the need for appropriate foundational research to identify target population needs and understand the capacities of the current health and related systems to inform the design of effective integrated strategies. Participants also expressed concerns about the limited evidence on implementation science outcomes for integrating family planning services with other health and non-health services. Implementation research covering measures such as the coverage, adherence, feasibility, acceptability and sustainability of integrated programmes was indicated as key. This included diagnosing what influences implementation outcomes, assessing the efficiency and effectiveness of strategies, and ultimately, guiding the process of translating evidence into practice. Participants emphasised the need for evidence on integrated family planning and nutrition services in the ‘real world’. One participant said,

…the evidence [on integration programmes] is not so strong…we have not been able to test integration programmes clearly and show the results to governments to make the case that this is how various programmes can be integrated with nutrition across sectors.”

It was suggested that donors, governments, NGOs, UN agencies and other stakeholders should create a common language for integrated approaches and systematically document their added value, to effectively advance the agenda for integration.

## Discussion

Family planning and nutrition services have commonly been integrated with programmes for maternal, neonatal and child health.^24 25^ The integration of family planning with nutrition programmes, however, is often overlooked and, therefore, we explored this topic in this study.

Study participants advocated integrated services for various reasons, including evidence suggesting that preventing early, multiple and poorly spaced pregnancies positively affects maternal nutrition, pregnancy and long-term child outcomes. These opinions are consistent with the previous research on adolescent pregnancy or birth spacing and increased risk of adverse maternal and infant outcomes,^26^,^28^ and evidence regarding negative correlations between family size and maternal nutritional status.^29^ More recently, large-scale analyses conducted using Demographic and Health Survey data from over 34 countries have indicated that short birth intervals (<24 months) lead to increased risk of perinatal mortality, infant mortality and undernutrition in children.^30 31^ Another important reason for integration was the acknowledgement of benefits to beneficiaries in terms of increased access to services in both domains and reducing missed opportunities. Integrated programmes targeting both domains were thus thought to have greater value in enhancing family planning, nutrition and related outcomes.

This study indicates three overarching models for integrating family planning, nutrition and other services: strengthening the health system, using a life course approach and leveraging multisectoral collaborations. Integrating health services at the delivery level has been perceived as crucial for comprehensive health systems strengthening strategies in resource-limited settings where family planning has been integrated with various services with mixed success.^1932^,^38^ Stakeholder perspectives also highlight the challenge of funding single-disease programmes over integrated approaches.^39^ Overcoming such barriers and strengthening health systems to offer comprehensive services can optimise resources and address multiple concerns simultaneously. While vertical programmes offer advantages, moving towards horizontal integration would require proven methods to maintain or improve outcomes.^40^,^42^

This study explores facilitators and barriers to integrating family planning, nutrition and other health services. Critical facilitators include strengthening core healthcare services, capacity building, supportive supervision and incentivisation, while barriers encompass shortages of trained personnel, infrastructure deficiencies, funding limitations and stock-outs of family planning commodities.^39 43^ Coordinated leadership and supportive policy environments are emphasised as key facilitators, along with clear budgeting, reporting, transparency and accountability.^32^ Many of these challenges and solutions extend beyond integration efforts, presenting opportunities to enhance overall population health services. However, specific barriers such as siloed funding and training gaps underscore the need for targeted efforts to promote and advance integration initiatives.

Despite recognising the potential benefits, concerns persist regarding the integration of family planning and nutrition in some LMICs due to prevailing sociocultural contexts. Some participants highlighted apprehensions about incorporating family planning and nutrition services, especially among adolescents, citing fears of affecting service uptake due to contraception stigma. Identifying and addressing cultural and religious barriers to family planning uptake will be crucial in designing and testing integration approaches. Engagement with the community, religious leaders, policy-makers and service providers should be built into the design and piloting process of integrated interventions.

Several areas in which the evidence is limited were also highlighted. Strengthening the evidence base regarding the advantages of and effective models for approaches integrating family planning and nutrition was deemed critical to promoting the adoption of such efforts by governments, donors and implementing agencies. Quantifying the impact of family planning on nutrition and related outcomes—including effects on maternal and child health, and longer-term implications for human capital is essential.^44^ A systematic review and meta-analysis similarly highlighted limited and uncertain evidence on the potential benefits of hormonal IUDs in improving haemoglobin levels and reducing BMI among women in LMICs, emphasising the need for high-quality research to support integrated interventions.^16^ Additionally, the use of a cost–benefit analysis or risk–benefit analysis could also be helpful for funding agencies and government officials to determine the magnitude of the health outcomes and savings that could be achieved through the integration of family planning and nutrition services. Additionally, participants stressed the importance of focusing on implementation research regarding potentially effective integration models to ensure effective translation of evidence into practice. The importance of creating a common language to describe integrated approaches and document their added value through multidisciplinary and multisectoral research efforts was noted. High-quality, well-documented research assessing various measures of effectiveness and cost-effectiveness of approaches integrating nutrition and family planning alongside other domains is a key next step to guiding further action.

Several international and regional guidelines and initiatives emphasise the importance of integrating nutrition and family planning services into existing public health systems.^45^,^51^ While international guidelines may not necessarily translate into national policies or service delivery, efforts to integrate nutrition services into public health systems and combine family planning with other health services have shown successful integration is achievable. The current study indicates awareness and recognition of the potential value of bringing together family planning and nutrition services alongside others to improve population health and documents experiences of recent efforts in this regard. Perspectives shared in this study indicate that while potentially beneficial, further action and evidence is required to develop and promote the adoption of effective strategies for integration.

To our knowledge, this is the first such exercise to better understand the current work and opportunities in this regard. This study lays important groundwork for further investigation into potentially impactful ways in which services in family planning, nutrition and other domains could be streamlined to achieve better health and other outcomes for women of reproductive age and children. Our study involved a limited number of interviews with global and regional stakeholders, potentially limiting the scope of findings. Despite this, participants provided detailed information, which suggested saturation. Further insights from government officials, local funding agencies and programme planners, and beneficiaries would enhance future studies.

## Conclusions

This qualitative study highlights the perspectives on the critical links between family planning and nutrition, and the value of integrating programmes in these domains to enhance outcomes for both. In addition to highlighting key barriers to integrating family planning and nutrition services, this study also indicates important opportunities to leverage in this regard, including building stronger evidence to further the integration agenda. In all, the findings of this study pave the way for future work examining programmatic approaches to best use these synergies to improve nutrition, family planning and multiple other related outcomes among women of reproductive age and their children.

## supplementary material

10.1136/bmjgh-2024-015932 online supplemental file 1

## Data Availability

Data are available on reasonable request.
