# Supplementary material for: Perspectives on integrating family planning and nutrition: a qualitative study of stakeholders
Source: BMJ Glob Health. 2025 Jan 6;10(Suppl 1):e015932. doi: 10.1136/bmjgh-2024-015932 (PMC11749386; doi:10.1136/bmjgh-2024-015932)
Supplement: online supplemental file 1 [file bmjgh-10-Suppl_1-s001.pdf]

Supplementary File 1: Example questions for interview from interview guide

1. The organization and participant's programmatic experience in the areas of nutrition and family planning

1.1. What programmes does your organization have in the domains of nutrition and family planning?

1.2. Please outline any prior or current efforts being undertaken by your organization or you around integrating nutrition and family planning.
Probes:
- What was the motivation behind integrating the two fields of programming?
- We are interested in understanding the approach taken and strategies used to integrate these two domains of programming. Please tell us more about this.
- What were the major lessons from this exercise in integrating nutrition and family planning?
- What were the major enablers or facilitators in this integration exercise?
- What were the major barriers to the integration of nutrition and family planning services? What strategies proved to be useful in overcoming these barriers?
- How successful has this specific integration effort been, and on what fronts?

1.3. What is your organization's and/or your experience in integrating family planning with other domains such as sexual and reproductive health services, post abortion care, maternal and child health and HIV/STI services, or child services including immunization.

2. Current global discourse, promising practices, opportunities, and considerations

2.1. What are the current discourses and initiatives on the global, regional, or country levels for integrating family planning with nutrition, or any other related services?
Probes:
- What are key points that have been raised in global or regional discourse in support of integration of these two domains, or alternatively, in favor of keeping them separate?
- What evidence-based models are available for the integration of family planning and nutrition services?
- What are different programmes in health systems being accorded with different levels of priority? Which programmes receive high priority, and which receive low priority? How may this affect efforts to integrate nutrition and family planning programmes?
- In what way the health systems issues such as competing resources and power dynamics may have a bearing on integration efforts?
- What is the potential of integrated nutrition and family planning programmes to improve the reach and combined impact of both services?
- To what level have any other global, regional, or country-specific programme integration efforts been implemented on the ground? What are the key takeaways from such efforts?

2.2. What are some key opportunities that could be leveraged to start bringing together programmes in these two domains?
Probes:
- What key factors would help facilitate such action on global and regional agenda?
- Who would be the key stakeholders to leverage such opportunities?
- What might be some challenges in pursuing such opportunities?
- What are important evidence gaps need to be addressed to support and inform such efforts?

2.3. What aspects would you recommend considering in integrating nutrition and family planning programmes?
Probes:

- What key stages in the life course may be of particular importance to target or prioritize?
- What platforms, settings, or service points would be valuable to target individuals at any or specific timepoints?
- What are some of the key context-specific factors to consider when designing and delivering such interventions?
- What are the challenges in integrating or adapting integrated interventions across regions? What would be useful ways to address these challenges?
